# Molecular characterization of two novel intronic variants of *NIPBL* gene detected in unrelated Cornelia de Lange syndrome patients

**DOI:** 10.1186/s12881-018-0738-y

**Published:** 2019-01-03

**Authors:** Natalia Krawczynska, Jolanta Wierzba, Jacek Jasiecki, Bartosz Wasag

**Affiliations:** 1 Department of Biology and Medical Genetics, Medical University of Gdansk, 1 Debinki Street, 80-211, Gdansk, Poland; 2 Laboratory of Clinical Genetics, University Clinical Centre, Gdansk, Poland; 3 Department of Pediatrics, Hematology and Oncology, Medical University of Gdansk, Gdansk, Poland; 4 Department of General Nursery, Medical University of Gdansk, Gdansk, Poland; 5 Department of Pharmaceutical Microbiology, Medical University of Gdansk, Gdansk, Poland

**Keywords:** CdLS, Cornelia de Lange syndrome, NIPBL gene, Splice-site variants, Next generation sequencing

## Abstract

**Background:**

Cornelia de Lange syndrome (CdLS), a rare, multisystemic disorder, has been linked to genetic alterations in *NIPBL*, *SMC1A*, *SMC3*, *HDAC8,* and *RAD21* genes. Approximately 60% of CdLS patients harbor various *NIPBL* variants. Genetic changes predicted to affect *NIPBL* gene splicing represent 15% of all *NIPBL* genetic abnormalities. Yet, only a few studies have investigated the molecular consequences of such variants.

**Case presentation:**

This study reports two novel, intronic *NIPBL* genetic variants in unrelated CdLS patients with the characteristic phenotype. A c.6954 + 3A > C substitution and a c.5862 + 1delG deletion were identified, one of each, in a 6 year-old boy and 39 month-old girl. Further studies confirmed that both variants introduce premature termination codons, resulting in the formation of truncated proteins p.(Ser2255LeufsTer20) and p.(Leu1955Ter), respectively.

**Conclusion:**

Single nucleotide alterations located within the conserved splice-donor site of intronic regions of the *NIPBL* gene can give rise to a premature termination of the translation and cause significant changes in the sequence of mRNA transcripts and NIPBL protein structure and function. The latter underline development of Cornelia de Lange syndrome phenotype.

**Electronic supplementary material:**

The online version of this article (10.1186/s12881-018-0738-y) contains supplementary material, which is available to authorized users.

## Background

Cornelia de Lange Syndrome (CdLS; OMIM# 1227470, 300,590, 610,759, 614,701, and 300,882) is a rare, multisystemic disorder characterized by the facial dysmorphism, limb malformations, intellectual disability, and developmental delay [1–6]. Hirsutism, growth failure, gastrointestinal problems, and behavioral abnormalities are commonly observed. Less frequent symptoms include congenital heart defects, hearing loss, seizures, and dysfunctional behaviors such as self-injury and rejection of social interactions. Projected incidence of CdLS is 1 per 10- to 30-thousand live births, although this may be a miscalculation due to an underdiagnosis of CdLS with mild clinical presentation [6].

Genetic alterations of *NIPBL*, *SMC1A*, *SMC3*, *HDAC8*, and *RAD21* genes are believed to trigger the development of Cornelia de Lange syndrome. Approximately 60% of CdLS patients harbor genetic variants in the *NIPBL* gene, which encodes human delangin, a homolog of Drosophila *Nipped-B* and fungal *Scc2* protein of the cohesin complex [7]. The majority of pathogenic *NIPBL*variants are distinctively unique in individual patients. Genetic mutations affecting *SMC1A*, *SMC3*, *HDAC8,* and *RAD21* genes account for 10% of CdLS cases [8–11]. Pathogenic variants within these genes affect the cohesion complex involved in sister chromatid cohesion and transcriptional gene regulation [8–13]. However, in 30% of CdLS patients, no molecular alterations have been identified.

The *NIPBL* gene, located on the short arm of chromosome 5 at p13.2, is 189 kbp in length and contains 47 exons that can be alternatively spliced into three isoforms of 2804, 2697, and 1101 amino acids. The 2804 amino acid nuclear protein plays a role in the maintenance of proper chromatin structure and in the communication between promoters and transcriptional enhancers [7]. The genetic alterations predicted to affect *NIPBL* splicing represent about 15% of all known variants in the *NIPBL* gene [6, 14]. Nevertheless, the alterations functional consequences on the mRNA splicing process are often not characterized.

This study presents two novel intronic *NIPBL* variants and reveals their impact on *NIPBL* mRNA transcripts.

## Case presentation

Two unrelated patients (hereinafter referred to as Individual 1 and Individual 2), who met the clinical criteria for a classic subtype of Cornelia de Lange syndrome, were enrolled in the study [1]. Both cases were diagnosed at the Department of Pediatrics, Hematology and Oncology, Medical University of Gdansk. The study was approved by the institutional review board (NKEBN/395/2014; NKEBN/395–504/2014; NKEBN/395–288/2014). Written informed consent to participate in the study was obtained from the patients’ parents.

Detailed clinical data are presented in Table 1. Individual 1 was a 6 year-old boy. He was born at 35 weeks of gestation. His birth length was 50 cm, weight 2100 g, and occipital frontal circumference 29 cm. Distinctive CdLS facial features have been presented, although no congenital malformations such as limb defects were seen. Presently, he exhibits self-injurious behavior, an attention deficit, and a learning disability.

**Table 1:** Clinical features of CdLS patients enrolled to the study

		Individual 1 CdLS24	Individual 2 CdLS62
	Age	6 years	3 months
	Gender	Male	Female
System	Specific feature		
Facial dysmorphy	Synophrys	+	+
	Long eyelashes	+	+
	Short nose	+	+
	Anteverted nostrils	+	+
	Long philtrum	+	+
	Broad or depressed nasal bridge	+	+
	Thin lips with downturned corners	+	+
	Cleft palate	+	+
	Low set ears	+	+
Growth	Birth weight (pregnancy week)	2100 g (35 hbd)	1640 g (39 hbd)
	Birth length	50 cm	44 cm
	Birth head circumference	29 cm	27,5 cm
	Postnatal microsomy	+	+
Development	Developmental delays or intellectual disability	+	Ne
	Learning disability	+	Ne
Behavior	Attention deficit disorder	+	Ne
	Hyperactivity	+	Ne
	Self-injurious behavior	+	Ne
	Autistic-like features	–	Ne
Musculoskeletal	Absent arms or forearms	–	Right hand ectrodactyly
	5th finger clinodactyly	+	+
	Abnormal palmar crease	+	+
	Short 1st knuckle/proximally placed thumb	+	+
Cardiac	Type of malformation	Ventricular septal defect	Tetralogy of Fallot
Gastrointestinal	Poor feeding	+	+
	Gastroesophageal reflux	+	+
Skin	Hirsutism	+	+
	Cutis marmorata	+	+
Neurologic	Seizures	–	–
	Hypertony	+	+
	Deafness or hearing loss	+	+
	Central nervous system malformation	–	Dandy Walker malformation
Uro genital	Cryptorchidism	+	–

+ feature present, − feature not present, Ne feature not evaluated

Individual 2 was a 3 month-old girl. She was born at 39 weeks of gestation. Her birth length was 44 cm, weight 1640 g, and occipital frontal circumference 27.5 cm. In addition to the distinctive CdLS facial features, she had a Dandy Walker malformation, tetralogy of Fallot, and right hand ectrodactyly. Moreover, feeding difficulties and gastroesophageal reflux were diagnosed.

Genomic DNA was extracted from peripheral blood samples using the Genomic DNA From Blood Kit (Macherey-Nagel) as per the manufacturer’s protocol. The quantity and quality of isolated DNA was determined with NanoDrop ND-1000 and Qubit Fluoremeter (Thermo Fisher Scientific).

Mutational analysis was performed using NimbleGen Seqcap EZ HyperCap (Roche Diagnostics) and MiSeq (Illumina). Probes were designed to enrich exons and 25 bp of flanking introns. The entire coding sequences of the following genes have been tested: *NIPBL*, *SMC1A*, *SMC3*, *RAD21*, *HDAC8*, *STAG1*, *SGOL1*, *PDS5A*, *PTTG1*, *TAF6*, *ESCO2*, *WAPAL*, *CDCA5*, *KMT2A*, *DDX11*,*ESPL1*, *PDS5B*, *PLK1*, *AURKB*, *ESCO1*, *MAU2*, *ATRX*, *STAG2*, and *RECQL4*. The read length was pair-end and a cut-off of 30% for the Variant Allele Frequency was applied. The analysis was performed using IGV (Broad Institute) and Alamut (Interactive Biosoftware) software. The nomenclature of the alterations was based on *NIPBL* mRNA sequence NM_133433.3, in accordance with the recommendations of the Human Genome Variation Society (http://varnomen.hgvs.org/) [15]. Variants’ pathogenicity was classified based on the American College of Medical Genetics (ACMG) guidelines [16]. The presence of *NIPBL* alterations was confirmed by an independent PCR followed by bidirectional Sanger sequencing. For Individual 1, exon-intron boundaries were amplified using NIPBL_F_40 (5′-TGTGTGTTTATCCTTTGCTTGC-3′) and NIPBL_R_40 (5′-GGACTAAGACTCCACCCTGTTG-3′) primes. Exon 32 of *NIPBL* gene of Individual 2 was amplified with NIPBL_F_32 (5′-GGCTAAAGCATAACAAAAGTATATT-3′) and NIPBL_R_32 (5′-AATAAATTTTCCCTACCAAAAGAA-3′) primers. PCR products were sequenced using a BigDye Terminator v3.1 Cycle Sequencing Kit and 3500 Series Genetic Analyzer (Thermo Fisher Scientific). Electropherograms were analyzed with Sequencer v.10 DNA Software (Gene Codes). Finally, both parents were genotyped in order to confirm de novo status of detected *NIPBL* variants.

Total RNA was extracted from blood samples using the Tempus Spin RNA Isolation Reagent Kit (Thermo Fisher Scientific). iScript cDNA Synthesis Kit (Bio-Rad) was applied for cDNA synthesis. For Individual 1, exons 38–42 of the *NIPBL* gene were amplified with cF38 (5′-AAGCTATCATTGGTCTAGGATTT-3′) and cR42 (5′-AGTCACGTCTGTTTTTGCTG-3′) primers. Exons 30–35 of the *NIPBL* gene of Individual 2 were amplified using cF30 (5′-ATGAAGAGGGCATTAAGAAATT-3′) and cR35 (5′-GAAATCATTTTGCGTACTAC-3′) primers. PCR products were excised from agarose gel, purified with the Gel Extraction KIT (Macherey-Nagel), and cloned into pGEM-T Easy Vector Systems (Promega). Plasmids were isolated using Plasmid Mini (A&A Biotechnology) and bidirectionally was sequenced with universal primers for pGEM-T Easy Vector.

**Fig. 1:**
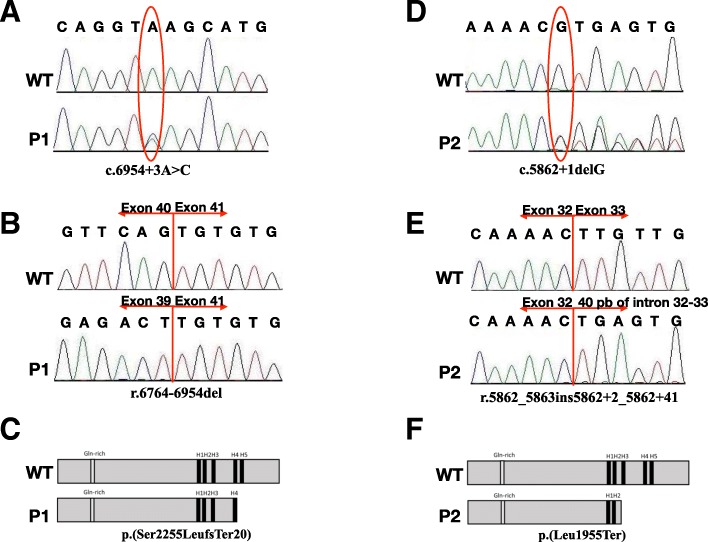
Variant analysis of both individuals. **a-c** Analysis of Individual 1 variant - c.6954 + 3A > C. **a** Sequence analysis of exon 40 and intron 40 of the *NIPBL* gene. The wild-type control (top panel) and Individual 1 (bottom panel). The mutation is marked with a circle. **b** Sequence analysis of cDNA *NIPBL* gene. The wild-type control (top panel) and the bottom panel exhibit an in-frame deletion of exon 40 of *NIPBL* gene in Individual 1. **c** NIPBL protein alignment of the wild-type control (top panel) and the bottom panel of Individual 1 exhibit protein truncation and loss of H5 domain. **d-f** Analysis of Individual 2 variant - c.5862 + 1delG. **d** Sequence analysis of exon 32 and intron 32 of the *NIPBL* gene. The wild-type control (top panel) and Individual 2 (bottom panel). The mutation is marked with a circle. **e** Sequence analysis of cDNA *NIPBL* gene. The wild-type control (top panel) and the bottom panel exhibit an in-frame insertion of 40 nucleotides of intron 32 of *NIPBL* gene in Individual 2. **f** NIPBL protein alignment of the wild-type control (top panel) and he bottom panel of Individual 2 exhibit protein truncation and loss of H3, H4, and H5 domains

Mutational analysis of two CdLS patients performed by NGS revealed the presence of novel, heterozygous, intronic variants in the NIPBL gene. In Individual 1, a c.6954 + 3A > C substitution was detected, while in Individual 2, a c.5862 + 1delG deletion was found. Molecular analysis performed on individuals’ parents confirmed de novo status of both alterations. Additional molecular studies performed with cDNA confirmed that both variants altered the splicing process. In Individual 1, intronic variant c.6954 + 3A > C caused r.6764_6954del on the mRNA level. In Individual 2, c.5862 + 1delG deletion resulted in r.5862_5863ins5862 + 2_5862 + 41 insertion on the mRNA level. In both cases, according to protein alignment, the protein was shortened (see Additional file 1). In Individual 1, the protein consisted of 2274 aa (amino acids) and the last 20 aa were changed - p.(Ser2255LeufsTer20). In Individual 2, a termination codon was introduced at codson 1955 - p.(Leu1955Ter). In both patients, highly conserved HEAT domains were disrupted. The results of the mutational analysis and predicted proteins are presented at Fig. 1.

## Discussion and conclusion

In this study, two novel, pathogenic variants affecting the conserved splice-donor site in the intronic regions of the *NIPBL* gene have been reported. Both alterations resulted in aberrant pre-mRNA splicing and caused the alteration of highly conserved HEAT domains. In Individual 1, the change of 20 aa (2255–2274) containing 13 aa of H4 domain and complete deletion of H5 domain has been identified. In Individual 2, deletion c.5862 + 1delG resulted in partial loss of the H3 domain (from 1954 aa) and complete deletion of H4 and H5 domains. As a result, the NIPBL ability to interact with other proteins could be severely damaged in both CdLS patients.

There are five HEAT repeats, H1-H5, located within the C-terminal part of the NIPBL protein. These domains, particularly H2 and H3, play a significant role in the interaction with histone deacetylases 1 and 3 (HDAC1 and HDAC3) and may initiate chromatin-remodeling processes [17, 18]. In general, HEAT repeats have been found in various chromosome-associated proteins, which probably play a role in chromosome dynamics [19].

Genetic alterations such as frame shift mutations, affecting both RNA transcripts and proteins have been identified in CdLS patients with severe phenotypes. In contrast, point mutations, not affecting the reading frame, were generally seen in patients with mild phenotype [6]. Individual 1, presented in this study, showed signs and symptoms seen in a severe CdLS phenotype, although no significant musculoskeletal abnormalities existed. This individual carried a c.6954 + 3A > C substitution in intron 40 of the *NIPBL* gene. Molecular alterations located within exons 35–47 of the *NIPBL* gene have been linked to a mild CdLS phenotype by a previously published investigation [6]. Individual 2 showed a spectrum of signs and symptoms seen in very severe phenotypes, including microsomy, severe limb and cardiac abnormalities, postnatal retardation, and prominent CdLS facial appearance. The more severe CdLS phenotype in Individual 2 can be explained by molecular findings. A c.5862 + 1delG deletion carried by Individual 2 resulted in an expression of a more dysfunctional NIPBL protein than in Individual 1.

Individual 2 suffers from tetralogy of Fallot (ToF). In general, this type of cardiac abnormality is rarely seen in CdLS patients (approximately 1%) [20]. One study reported three patients with ToF in a group of 310 CdLS individuals [21]. In another study, based on an Asian population, only one individual with ToF was observed among 50 patients [22]. EUROCAT studies identified two patients with ToF among 106 CdLS clinical cases [23]. In general, tetralogy of Fallot was diagnosed in CdLS patients with severe phenotypes carrying different *NIPBL* variants including substitutions, in-frame deletions, or truncating variants. However, due to the limited number of CdLS patients with ToF, it is impossible to correlate this clinical feature with molecular data. Nevertheless, a girl with a severe, classical type of Cornelia de Lange syndrome was diagnosed with Fallot’s tetralogy and a large deletion encompassing exons 41–42 of the *NIPBL* gene (deletion expanding from intron 40 to part of intron 42 - c.6955–1095 _7263 + 3344del5227) [24]. Also, the case of an 11 month-old CdLS girl with ToF and double lumen aortic arch has been reported; however, the molecular status of her *NIPBL* was unknown [25]. In the current study, in Individual 2 (with ToF), an insertion of 40 bp of intron 32 of NIPBL transcript was identified. Other types of cardiovascular disorders are not a major criteria for CdLS, although occur fairly often [20–26].

Congenital brain defects can be observed in CdLS patients, however they seem to be extremely rare. Individual 2 was diagnosed with the Dandy Walker malformation, a rare malformation that involves the cerebellum and fourth ventricle [27]. The Dandy Walker malformation has not been reported in CdLS patients.

It is accepted that genetic variants located at the exon-intron junctions of genes affect the splicing process, changing the sequence and structure of the protein and impairing its function. Therefore, such intronic variants can be definitely classified as pathogenic and responsible for the severe phenotypes of the patients [18]. In the current study, we have presented two CdLS patients with novel, intronic *NIPBL* variants affecting both transcription and translation. These results underline the necessity for a comprehensive molecular analysis of intronic variants both on the DNA and RNA levels. Such studies can not only give new insights into the molecular mechanism of the disease, but may also allow us to perform a more detailed genotype-phenotype correlation in a group of CdLS patients.

In summary, we have described two unrelated patients with Cornelia de Lange syndrome caused by de novo splice-site mutations in the *NIPBL* gene. In both individuals, the detected variants lead to a production of aberrant mRNA transcripts encoding truncated NIPBL proteins. In Individual 1 (c.6954 + 3A > C; r.6764_6954del), the protein was shortened by 423 aa, with the last 20 aa changed. In Individual 2 (c.5862 + 1delG; r.5862_5863ins5862 + 2_5862 + 41), deletion of 743 aa was observed. These findings highlight that single nucleotide alterations located within the conserved splice-donor site of the intronic regions of the *NIPBL* gene could have a severe impact on the sequence and structure of both mRNA transcripts and proteins. Given this, a comprehensive molecular analysis of such genetic variants should be considered.

## Additional file

Additional file 1: NIPBL protein alignment of both cases. NIPBL wt – first row – NIPBL alignment of wild type protein. NIPBL p1 – second row - NIPBL alignment of Individual 1 with c.6954 + 3A > C variant. NIPBL p2 – third row - NIPBL alignment of Individual 2 with c.5862 + 1delG variant. (PDF 154 kb)

## Abbreviations

aa: Amino acids
CdLS: Cornelia de Lange syndrome
DNA: Deoxyribonucleic acid
hbd: Pregnancy week
HEAT: Huntington, Elongation factor 3, the PR65/A subunit of PP2A and the lipid kinase Tor
IGV: Integrative Genomics Viewer
mRNA: messenger ribonucleic acid
ng: Nanogram
NGS: Next generation sequencing
NIPBL: Nipped-B-like protein
pb: Pars of bases
RNA: Ribonucleic acid
ToF: Tetralogy of Fallot
