# Supplementary material for: Molecular characterization of two novel intronic variants of NIPBL gene detected in unrelated Cornelia de Lange syndrome patients
Source: BMC Med Genet. 2019 Jan 3;20:1. doi: 10.1186/s12881-018-0738-y (PMC6318863; doi:10.1186/s12881-018-0738-y)
Supplement: Supplementary file 1 — NIPBL protein alignment of both cases. NIPBL wt – first row – NIPBL alignment of wild type protein. NIPBL p1 – second row - NIPBL alignment of Individual 1 with c.6954 + 3A > C variant. NIPBL p2 – third row - NIPBL alignment of Individual 2 with c.5862 + 1delG variant. (PDF 154 kb) [file 12881_2018_738_MOESM1_ESM.pdf]

```
                   10         20         30         40         50         60         70         80         90        100
           ....|....| ....|....| ....|....| ....|....| ....|....| ....|....| ....|....| ....|....| ....|....| ....|....|
NIPBL wt   MNGDMPHVPI TTLAGIASLT DLLNQLPLPS PLPATTTKSL LFNARIAEEV NCLLACRDDN LVSQLVHSLN QVSTDHIELK DNLGSDDPEG DIPVLLQAVL
NIPBL P1   .......... .......... .......... .......... .......... .......... .......... .......... .......... ..........
NIPBL P2   .......... .......... .......... .......... .......... .......... .......... .......... .......... ..........

                  110        120        130        140        150        160        170        180        190        200
           ....|....| ....|....| ....|....| ....|....| ....|....| ....|....| ....|....| ....|....| ....|....| ....|....|
NIPBL wt   ARSPNVFREK SMQNRYVQSG MMMSQYKLSQ NSMHSSPASS NYQQTTISHS PSSRFVPPQT SSGNRFMPQQ NSPVPSPYAP QSPAGYMPYS HPSSYTTHPQ
NIPBL P1   .......... .......... .......... .......... .......... .......... .......... .......... .......... ..........
NIPBL P2   .......... .......... .......... .......... .......... .......... .......... .......... .......... ..........

                  210        220        230        240        250        260        270        280        290        300
           ....|....| ....|....| ....|....| ....|....| ....|....| ....|....| ....|....| ....|....| ....|....| ....|....|
NIPBL wt   MQQASVSSPI VAGGLRNIHD NKVSGPLSGN SANHHADNPR HGSSEDYLHM VHRLSSDDGD SSTMRNAASF PLRSPQPVCS PAGSEGTPKG SRPPLILQSQ
NIPBL P1   .......... .......... .......... .......... .......... .......... .......... .......... .......... ..........
NIPBL P2   .......... .......... .......... .......... .......... .......... .......... .......... .......... ..........

                  310        320        330        340        350        360        370        380        390        400
           ....|....| ....|....| ....|....| ....|....| ....|....| ....|....| ....|....| ....|....| ....|....| ....|....|
NIPBL wt   SLPCSSPRDV PPDILLDSPE RKQKKQKKMK LGKDEKEQSE KAAMYDIISS PSKDSTKLTL RLSRVRSSDM DQQEDMISGV ENSNVSENDI PFNVQYPGQT
NIPBL P1   .......... .......... .......... .......... .......... .......... .......... .......... .......... ..........
NIPBL P2   .......... .......... .......... .......... .......... .......... .......... .......... .......... ..........

                  410        420        430        440        450        460        470        480        490        500
           ....|....| ....|....| ....|....| ....|....| ....|....| ....|....| ....|....| ....|....| ....|....| ....|....|
NIPBL wt   SKTPITPQDI NRPLNAAQCL SQQEQTAFLP ANQVPVLQQN TSVAAKQPQT SVVQNQQQIS QQGPIYDEVE LDALAEIERI ERESAIERER FSKEVQDKDK
NIPBL P1   .......... .......... .......... .......... .......... .......... .......... .......... .......... ..........
NIPBL P2   .......... .......... .......... .......... .......... .......... .......... .......... .......... ..........

                  510        520        530        540        550        560        570        580        590        600
           ....|....| ....|....| ....|....| ....|....| ....|....| ....|....| ....|....| ....|....| ....|....| ....|....|
NIPBL wt   PLKKRKQDSY PQEAGGATGG NRPASQETGS TGNGSRPALM VSIDLHQAGR VDSQASITQD SDSIKKPEEI KQCNDAPVSV LQEDIVGSLK STPENHPETP
NIPBL P1   .......... .......... .......... .......... .......... .......... .......... .......... .......... ..........
NIPBL P2   .......... .......... .......... .......... .......... .......... .......... .......... .......... ..........

                  610        620        630        640        650        660        670        680        690        700
           ....|....| ....|....| ....|....| ....|....| ....|....| ....|....| ....|....| ....|....| ....|....| ....|....|
NIPBL wt   KKKSDPELSK SEMKQSESRL AESKPNENRL VETKSSENKL ETKVETQTEE LKQNESRTTE CKQNESTIVE PKQNENRLSD TKPNDNKQNN GRSETTKSRP
NIPBL P1   .......... .......... .......... .......... .......... .......... .......... .......... .......... ..........
NIPBL P2   .......... .......... .......... .......... .......... .......... .......... .......... .......... ..........

                  710        720        730        740        750        760        770        780        790        800
           ....|....| ....|....| ....|....| ....|....| ....|....| ....|....| ....|....| ....|....| ....|....| ....|....|
NIPBL wt   ETPKQKGESR PETPKQKSDG HPETPKQKGD GRPETPKQKG ESRPETPKQK NEGRPETPKH RHDNRRDSGK PSTEKKPEVS KHKQDTKSDS PRLKSERAEA
NIPBL P1   .......... .......... .......... .......... .......... .......... .......... .......... .......... ..........
NIPBL P2   .......... .......... .......... .......... .......... .......... .......... .......... .......... ..........

                  810        820        830        840        850        860        870        880        890        900
           ....|....| ....|....| ....|....| ....|....| ....|....| ....|....| ....|....| ....|....| ....|....| ....|....|
NIPBL wt   LKQRPDGRSV SESLRRDHDN KQKSDDRGES ERHRGDQSRV RRPETLRSSS RNEHGIKSDS SKTDKLERKH RHESGDSRER PSSGEQKSRP DSPRVKQGDS
NIPBL P1   .......... .......... .......... .......... .......... .......... .......... .......... .......... ..........
NIPBL P2   .......... .......... .......... .......... .......... .......... .......... .......... .......... ..........

                  910        920        930        940        950        960        970        980        990       1000
           ....|....| ....|....| ....|....| ....|....| ....|....| ....|....| ....|....| ....|....| ....|....| ....|....|
NIPBL wt   NKSRSDKLGF KSPTSKDDKR TEGNKSKVDT NKAHPDNKAE FPSYLLGGRS GALKNFVIPK IKRDKDGNVT QETKKMEMKG EPKDKVEKIG LVEDLNKGAK
NIPBL P1   .......... .......... .......... .......... .......... .......... .......... .......... .......... ..........
NIPBL P2   .......... .......... .......... .......... .......... .......... .......... .......... .......... ..........

                 1010       1020       1030       1040       1050       1060       1070       1080       1090       1100
           ....|....| ....|....| ....|....| ....|....| ....|....| ....|....| ....|....| ....|....| ....|....| ....|....|
NIPBL wt   PVVVLQKLSL DDVQKLIKDR EDKSRSSLKP IKNKPSKSNK GSIDQSVLKE LPPELLAEIE STMPLCERVK MNKRKRSTVN EKPKYAEISS DEDNDSDEAF
NIPBL P1   .......... .......... .......... .......... .......... .......... .......... .......... .......... ..........
NIPBL P2   .......... .......... .......... .......... .......... .......... .......... .......... .......... ..........

                 1110       1120       1130       1140       1150       1160       1170       1180       1190       1200
           ....|....| ....|....| ....|....| ....|....| ....|....| ....|....| ....|....| ....|....| ....|....| ....|....|
NIPBL wt   ESSRKRHKKD DDKAWEYEER DRRSSGDHRR SGHSHEGRRS SGGGRYRNRS PSDSDMEDYS PPPSLSEVAR KMKKKEKQKK RKAYEPKLTP EEMMDSSTFK
NIPBL P1   .......... .......... .......... .......... .......... .......... .......... .......... .......... ..........
NIPBL P2   .......... .......... .......... .......... .......... .......... .......... .......... .......... ..........

                 1210       1220       1230       1240       1250       1260       1270       1280       1290       1300
           ....|....| ....|....| ....|....| ....|....| ....|....| ....|....| ....|....| ....|....| ....|....| ....|....|
NIPBL wt   RFTASIENIL DNLEDMDFTA FGDDDEIPQE LLLGKHQLNE LGSESAKIKA MGIMDKLSTD KTVKVLNILE KNIQDGSKLS TLLNHNNDTE EEERLWRDLI
NIPBL P1   .......... .......... .......... .......... .......... .......... .......... .......... .......... ..........
NIPBL P2   .......... .......... .......... .......... .......... .......... .......... .......... .......... ..........

                 1310       1320       1330       1340       1350       1360       1370       1380       1390       1400
           ....|....| ....|....| ....|....| ....|....| ....|....| ....|....| ....|....| ....|....| ....|....| ....|....|
NIPBL wt   MERVTKSADA CLTTINIMTS PNMPKAVYIE DVIERVIQYT KFHLQNTLYP QYDPVYRLDP HGGGLLSSKA KRAKCSTHKQ RVIVMLYNKV CDIVSSLSEL
NIPBL P1   .......... .......... .......... .......... .......... .......... .......... .......... .......... ..........
NIPBL P2   .......... .......... .......... .......... .......... .......... .......... .......... .......... ..........

                 1410       1420       1430       1440       1450       1460       1470       1480       1490       1500
           ....|....| ....|....| ....|....| ....|....| ....|....| ....|....| ....|....| ....|....| ....|....| ....|....|
NIPBL wt   LEIQLLTDTT ILQVSSMGIT PFFVENVSEL QLCAIKLVTA VFSRYEKHRQ LILEEIFTSL ARLPTSKRSL RNFRLNSSDM DGEPMYIQMV TALVLQLIQC
NIPBL P1   .......... .......... .......... .......... .......... .......... .......... .......... .......... ..........
```

```
NIPBL P2   .......... .......... .......... .......... .......... .......... .......... .......... .......... ..........

                    1510       1520       1530       1540       1550       1560       1570       1580       1590       1600
           ....|....| ....|....| ....|....| ....|....| ....|....| ....|....| ....|....| ....|....| ....|....| ....|....|
NIPBL wt   VVHLPSSEKD SNAEEDSNKK IDQDVVITNS YETAMRTAQN FLSIFLKKCG SKQGEEDYRP LFENFVQDLL STVNKPEWPA AELLLSLLGR LLVHQFSNKS
NIPBL P1   .......... .......... .......... .......... .......... .......... .......... .......... .......... ..........
NIPBL P2   .......... .......... .......... .......... .......... .......... .......... .......... .......... ..........

                    1610       1620       1630       1640       1650       1660       1670       1680       1690       1700
           ....|....| ....|....| ....|....| ....|....| ....|....| ....|....| ....|....| ....|....| ....|....| ....|....|
NIPBL wt   TEMALRVASL DYLGTVAARL RKDAVTSKMD QGSIERILKQ VSGGEDEIQQ LQKALLDYLD ENTETDPSLV FSRKFYIAQW FRDTTLETEK AMKSQKDEES
NIPBL P1   .......... .......... .......... .......... .......... .......... .......... .......... .......... ..........
NIPBL P2   .......... .......... .......... .......... .......... .......... .......... .......... .......... ..........

                    1710       1720       1730       1740       1750       1760       1770       1780       1790       1800
           ....|....| ....|....| ....|....| ....|....| ....|....| ....|....| ....|....| ....|....| ....|....| ....|....|
NIPBL wt   SEGTHHAKEI ETTGQIMHRA ENRKKFLRSI IKTTPSQFST LKMNSDTVDY DDACLIVRYL ASMRPFAQSF DIYLTQILRV LGENAIAVRT KAMKCLSEVV
NIPBL P1   .......... .......... .......... .......... .......... .......... .......... .......... .......... ..........
NIPBL P2   .......... .......... .......... .......... .......... .......... .......... .......... .......... ..........

                    1810       1820       1830       1840       1850       1860       1870       1880       1890       1900
           ....|....| ....|....| ....|....| ....|....| ....|....| ....|....| ....|....| ....|....| ....|....| ....|....|
NIPBL wt   AVDPSILARL DMQRGVHGRL MDNSTSVREA AVELLGRFVL CRPQLAEQYY DMLIERILDT GISVRKRVIK ILRDICIEQP TFPKITEMCV KMIRRVNDEE
NIPBL P1   .......... .......... .......... .......... .......... .......... .......... .......... .......... ..........
NIPBL P2   .......... .......... .......... .......... .......... .......... .......... .......... .......... ..........

                    1910       1920       1930       1940       1950       1960       1970       1980       1990       2000
           ....|....| ....|....| ....|....| ....|....| ....|....| ....|....| ....|....| ....|....| ....|....| ....|....|
NIPBL wt   GIKKLVNETF QKLWFTPTPH NDKEAMTRKI LNITDVVAAC RDTGYDWFEQ LLQNLLKSEE DSSYKPVKKA CTQLVDNLVE HILKYEESLA DSDNKGVNSG
NIPBL P1   .......... .......... .......... .......... .......... .......... .......... .......... .......... ..........
NIPBL P2   .......... .......... .......... .......... .......... ....------ ---------- ---------- ---------- ----------

                    2010       2020       2030       2040       2050       2060       2070       2080       2090       2100
           ....|....| ....|....| ....|....| ....|....| ....|....| ....|....| ....|....| ....|....| ....|....| ....|....|
NIPBL wt   RLVACITTLF LFSKIRPQLM VKHAMTMQPY LTTKCSTQND FMVICNVAKI LELVVPLMEH PSETFLATIE EDLMKLIIKY GMTVVQHCVS CLGAVVNKVT
NIPBL P1   .......... .......... .......... .......... .......... .......... .......... .......... .......... ..........
NIPBL P2   ---------- ---------- ---------- ---------- ---------- ---------- ---------- ---------- ---------- ----------

                    2110       2120       2130       2140       2150       2160       2170       2180       2190       2200
           ....|....| ....|....| ....|....| ....|....| ....|....| ....|....| ....|....| ....|....| ....|....| ....|....|
NIPBL wt   QNFKFVWACF NRYYGAISKL KSQHQEDPNN TSLLTNKPAL LRSLFTVGAL CRHFDFDLED FKGNSKVNIK DKVLELLMYF TKHSDEEVQT KAIIGLGFAF
NIPBL P1   .......... .......... .......... .......... .......... .......... .......... .......... .......... ..........
NIPBL P2   ---------- ---------- ---------- ---------- ---------- ---------- ---------- ---------- ---------- ----------

                    2210       2220       2230       2240       2250       2260       2270       2280       2290       2300
           ....|....| ....|....| ....|....| ....|....| ....|....| ....|....| ....|....| ....|....| ....|....| ....|....|
NIPBL wt   IQHPSLMFEQ EVKNLYNNIL SDKNSSVNLK IQVLKNLQTY LQEEDTRMQQ ADRDWKKVAK QEDLKEMGDV SSGMSSSIMQ LYLKQVLEAF FHTQSSVRHF
NIPBL P1   .......... .......... .......... .......... .......... ....LCAIFN CYGHRPRTCY AEQG------ ---------- ----------
NIPBL P2   ---------- ---------- ---------- ---------- ---------- ---------- ---------- ---------- ---------- ----------

                    2310       2320       2330       2340       2350       2360       2370       2380       2390       2400
           ....|....| ....|....| ....|....| ....|....| ....|....| ....|....| ....|....| ....|....| ....|....| ....|....|
NIPBL wt   ALNVIALTLN QGLIHPVQCV PYLIAMGTDP EPAMRNKADQ QLVEIDKKYA GFIHMKAVAG MKMSYQVQQA INTCLKDPVR GFRQDESSSA LCSHLYSMIR
NIPBL P1   ---------- ---------- ---------- ---------- ---------- ---------- ---------- ---------- ---------- ----------
NIPBL P2   ---------- ---------- ---------- ---------- ---------- ---------- ---------- ---------- ---------- ----------

                    2410       2420       2430       2440       2450       2460       2470       2480       2490       2500
           ....|....| ....|....| ....|....| ....|....| ....|....| ....|....| ....|....| ....|....| ....|....| ....|....|
NIPBL wt   GNRQHRRAFL ISLLNLFDDT AKTDVTMLLY IADNLACFPY QTQEEPLFIM HHIDITLSVS GSNLLQSFKE SMVKDKRKER KSSPSKENES SDSEEEVSRP
NIPBL P1   ---------- ---------- ---------- ---------- ---------- ---------- ---------- ---------- ---------- ----------
NIPBL P2   ---------- ---------- ---------- ---------- ---------- ---------- ---------- ---------- ---------- ----------

                    2510       2520       2530       2540       2550       2560       2570       2580       2590       2600
           ....|....| ....|....| ....|....| ....|....| ....|....| ....|....| ....|....| ....|....| ....|....| ....|....|
NIPBL wt   RKSRKRVDSD SDSDSEDDIN SVMKCLPENS APLIEFANVS QGILLLLMLK QHLKNLCGFS DSKIQKYSPS ESAKVYDKAI NRKTGVHFHP KQTLDFLRSD
NIPBL P1   ---------- ---------- ---------- ---------- ---------- ---------- ---------- ---------- ---------- ----------
NIPBL P2   ---------- ---------- ---------- ---------- ---------- ---------- ---------- ---------- ---------- ----------

                    2610       2620       2630       2640       2650       2660       2670       2680       2690       2700
           ....|....| ....|....| ....|....| ....|....| ....|....| ....|....| ....|....| ....|....| ....|....| ....|....|
NIPBL wt   MANSKITEEV KRSIVKQYLD FKLLMEHLDP DEEEEEGEVS ASTNARNKAI TSLLGGGSPK NNTAAETEDD ESDGEDRGGG TSGSLRRSKR NSDSTELAAQ
NIPBL P1   ---------- ---------- ---------- ---------- ---------- ---------- ---------- ---------- ---------- ----------
NIPBL P2   ---------- ---------- ---------- ---------- ---------- ---------- ---------- ---------- ---------- ----------

                    2710       2720       2730       2740       2750       2760       2770       2780       2790       2800
           ....|....| ....|....| ....|....| ....|....| ....|....| ....|....| ....|....| ....|....| ....|....| ....|....|
NIPBL wt   MNESVDVMDV IAICCPKYKD RPQIARVVQK TSSGFSVQWM AGSYSGSWTE AKRRDGRKLV PWVDTIKESD IIYKKIALTS ANKLTNKVVQ TLRSLYAAKD
NIPBL P1   ---------- ---------- ---------- ---------- ---------- ---------- ---------- ---------- ---------- ----------
NIPBL P2   ---------- ---------- ---------- ---------- ---------- ---------- ---------- ---------- ---------- ----------


           ....
NIPBL wt   GTSS
NIPBL P1   ----
NIPBL P2   ----
```
